# Relationships between Psychosocial Resilience and Physical Health Status of Western Australian Urban Aboriginal Youth

**DOI:** 10.1371/journal.pone.0145382

**Published:** 2015-12-30

**Authors:** Katrina D. Hopkins, Carrington C. J. Shepherd, Catherine L. Taylor, Stephen R. Zubrick

**Affiliations:** Telethon Kids Institute, The University of Western Australia, Crawley, Western Australia, Australia; Queensland University of Technology, AUSTRALIA

## Abstract

**Background:**

Psychosocial processes are implicated as mediators of racial/ethnic health disparities via dysregulation of physiological responses to stress. Our aim was to investigate the extent to which factors previously documented as buffering the impact of high-risk family environments on Aboriginal youths’ psychosocial functioning were similarly beneficial for their physical health status.

**Method and Results:**

We examined the relationship between psychosocial resilience and physical health of urban Aboriginal youth (12–17 years, n = 677) drawn from a representative survey of Western Australian Aboriginal children and their families. A composite variable of psychosocial resilient status, derived by cross-classifying youth by high/low family risk exposure and normal/abnormal psychosocial functioning, resulted in four groups- Resilient, Less Resilient, Expected Good and Vulnerable. Separate logistic regression modeling for high and low risk exposed youth revealed that Resilient youth were significantly more likely to have lower self-reported asthma symptoms (OR 3.48, p<.001) and carer reported lifetime health problems (OR 1.76, p<.04) than Less Resilient youth.

**Conclusion:**

The findings are consistent with biopsychosocial models and provide a more nuanced understanding of the patterns of risks, resources and adaptation that impact on the physical health of Aboriginal youth. The results support the posited biological pathways between chronic stress and physical health, and identify the protective role of social connections impacting not only psychosocial function but also physical health. Using a resilience framework may identify potent protective factors otherwise undetected in aggregated analyses, offering important insights to augment general public health prevention strategies.

## Introduction

Despite comprising a small proportion of the total Australian population (3% or around 670,000), Australian Aboriginal and Torres Strait Islander peoples (the Indigenous peoples of the Australian continent and referred to hereafter as ‘Aboriginal’) experience pervasive disparities across multiple indices of health and wellbeing [1]. These disparities have their origins in, and reflect the downstream impact of, colonial policies imposing forced removal of Aboriginal people from their land and their children from their natural families [2]. Now, the current generation of Aboriginal young people experience the impact of these historical policies through more proximal exposures to related but different sets of risks that include family dysfunction, alcohol abuse, and incarceration [3, 4]. This is indexed in a life expectancy that is 9–11 years lower than that of the Australian non-Aboriginal population with 68% of deaths in 2006–10 due to chronic diseases such as circulatory disease, diabetes and respiratory disease [1].

Low SES and its accompanying multiple and unmitigated stresses is strongly implicated in this pathway [5, 6]. Exposure to “levels of stress associated with excessive, persistent, and/or uncontrollable adversity, without the buffering protection of stable adult support, is associated with disruptive effects on multiple organ systems that can lead to lifelong disease” [7]. Exposure to chronic stress has been linked to physiologic dysregulation of the hypothalamic-pituitary-adrenal (HPA) [8] and other metabolic and immune systems [9–11], and premature poor health and aging [12–15].

### Low SES, Psychological Appraisal of Chronic Stress and Asthma

Processes involving cognitive and emotional appraisal of stressors and the triggering of physiological responses are increasingly implicated as mediating the ubiquitous relationship between low SES and a range of physical health outcomes [16, 17]. These outcomes include cardiovascular disease and asthma symptoms specifically [18–21].

One pathway from low SES to asthma involves the negative appraisal of, and physiological responses to, chronic stress and consequential heightened inflammatory profiles indicated by elevated c-reactive protein and interleukin-6 [18, 22, 23]. In both minority and Indigenous populations, for example, studies have found that racism [24, 25] and even the anticipation of discrimination or stereotype threat is associated with physiological arousal [26, 27].

Using a resilience framework to examine adaptive processes that ameliorate the impact of adverse environments on health outcomes may enable a more nuanced understanding of critical pathways entrenching or mitigating health inequalities. Gallo [28] proposes a “reserve capacity” model in which the interactions between SES, chronic stress, resilience resources and cognitive-emotional processing link low SES with poor health outcomes. Low SES individuals exposed to higher levels of chronic stress experience higher levels of negative emotionality, depression, anxiety and hostility, which in turn increased the likelihood of negative physiological and behavioral health responses leading to poorer health outcomes. Resilience resources (e.g., personal and social factors such as self-esteem, optimism and social support) are posited as potentially moderating the low SES-health relationship.

A quantitative resilience framework was used in our previous work in order to understand factors uniquely protecting the psychosocial functioning of Western Australian Aboriginal youth living in high risk family environments. Notably, this research identified 58% of young people as psychosocially resilient despite exposure to high risk family contexts, with prosocial peers protecting adverse outcomes for these youth [29].

The extent to which psychosocial risks are associated with physical health outcomes in the Australian Aboriginal context has received scant attention [30–32] and gives rise to a number of uncertainties in the current evidence base. First, the nature of risks and resources experienced by low SES non-minority populations may not be the same as those experienced by minority populations such as African Americans [33–35] and Australian Aboriginal youth specifically[1, 36]. Second, although the majority of the Australian Aboriginal population lives in urban environments the majority of research on physical health outcomes has been undertaken in remote locations [37]. Third, much of the health disparities research focuses on single risk exposures rather than the examining the net effect of multiple risks and resources [38–40]. Few studies have adopted multilevel ecological models that take into account multiple *and* context specific risks and protective factors influencing diseases [41], factors buffering the impact of allostatic load on physical health outcomes [42], nor how these factors may vary *within* the Australian Aboriginal population [43].

In this study, we examined whether specific configurations of risks and resources (or reserve capacity) protects some Aboriginal young people but not others from the adverse physical effects of psychosocial risks, with specific reference to symptoms of asthma. We focus on asthma symptoms specifically as the hospitalization rate for Aboriginal people is more than double that of other Australians, and urban Aboriginal youth are additionally at higher risk by virtue of their urban locality and low socioeconomic status [44]. We use a contemporary resilience framework of analysis to examine whether the factors that buffer the impact of high risk environments on psychosocial functioning of Aboriginal urban youth confer similar benefits for their physical health, as measured by primary carer reports of their lifetime health problems and youth reported asthma symptoms.

### Current study

Our current analysis extends previous work by Hopkins et al [29, 45] by hypothesizing that the social connections buffering the mental health of high risk exposed youth from the impact of high risk family environments will also confer protection for their physical health as measured by symptoms of asthma. In our previous work we used a composite variable of psychosocial resilient status to test the hypothesis that psychosocially resilient Aboriginal youth (Resilient) would have reported better physical health than less psychosocially resilient youth (Less Resilient). Because this variable is a key construct it is described more fully in the Methods and Table 1.

**Table 1:** Psychosocial Resilient Status Variable (N = 5180, 95% CI 5130, 5180)–a person based cross-classification of family risk context and psychosocial function [29, 45].

	Psychosocial Resilient Status Groups			
	Resilient	Less Resilient	Expected Good	Vulnerable
Prevalence	26.4%(22.6, 30.7)	21.4%(17.8, 25.5)	41.3%(36.6, 46.2)	10.9%(8.4, 13.8)
Family Risk Context	High		Low	
Psychosocial function	Good	Poor	Good	Poor
Factors associated with good psychosocial function^1^	Prosocial friend		No reported exposure to racism	
	Lower SES neighborhood		Higher self-esteem	
	Higher self-esteem		Higher self-regulation	
	Higher self-regulation			

^1^ Separate logistic regression models using the same set of predictor variables were conducted for subsets of youth in high and low family risk contexts. In high risk settings Resilient youth were more likely than Less Resilient youth to have these characteristics; and similarly, in low risk contexts Expected Good youth were more likely than Vulnerable youth to have these characteristics.

We include additional independent variables in the modeling: at the *individual level* of influence, e.g., proportion of optimal birth weight/gestational age [46, 47], cigarette smoking [48]; at the *family level*, e.g., parental cigarette smoking [19, 49], poor quality housing [19, 50]; and at the *neighborhood level*, e.g., a Census-based measure of neighborhood socioeconomic disadvantage [51, 52].

## Method

As the current study draws a sample of urban Aboriginal youth from data collected by the Western Australian Aboriginal Child Health Survey 2000–2002 (WAACHS) a brief overview of this epidemiological survey is provided.

The WAACHS implemented a state-wide area-based clustered multi-stage random sample design selected in three stages: census collection districts (CDs), households, and children. CDs were selected with the probability of inclusion proportional to the number of Aboriginal and Torres Strait Islander children living in the CD. The primary sampling unit for the WAACHS was households. Households were eligible to be selected for participation if they had (an) Aboriginal child(ren) aged 0–17 years. When households had more than one Aboriginal child in this age range all Aboriginal children were selected.

The WAACHS identified a random sample of 2,386 households with 6,209 Aboriginal children as eligible. Of this, 1,999 (84%) households agreed to participate. These 1,999 households had 5,513 children of whom 1,480 were youth aged 12–17 years. Parent-report data were secured on 1,480 youth and of these youth 1,073 (73%) provided self-report data. Parental consent and individual consent was obtained from all participants, and data were collected via face to face interviews and data linkage with State Government administrative datasets. The WAACHS Aboriginal Steering Committee gave direction to the cultural appropriateness of survey instruments and methodology. Details of the design and implementation of the WAACHS are described extensively elsewhere [53, 54] and can be accessed online at http://aboriginal.telethonkids.org.au/kulunga-research-network/waachs.aspx.

### Ethics

Human research ethics approval for this study was obtained from the Human Research Ethics Committee of The University of Western Australia (RA/4/1/4810) and the Western Australian Aboriginal Health Ethics Committee (formerly the Western Australian Aboriginal Health Information and Ethics Committee) of the Aboriginal Health Council of Western Australia (Ref 298–07.10). Cultural advice and guidance was obtained for this current study through the Western Australian Aboriginal Consultative Council Advising Research and Evaluation (ACCARE) based at the Telethon Kids Institute in Perth, Western Australia. The WAACHS was conducted under the direction of the project’s Aboriginal Steering Committee with extensive attention to the cultural appropriateness of survey instruments and methodology. The written permission of primary carers of Aboriginal children aged 0–17 years was obtained for data-linkage information to be collected, and for youth aged 12–17 years to be interviewed, as part of the WAACHS.

### Study Sample

The study sample for this paper was selected if they were 12–17 years old, had both Youth Self Report and primary carer reports, and lived in urbanized areas of WA such as the capital city of Perth or large regional centres (e.g., Kalgoorlie and Geraldton). A total of N = 677 met these criteria. Using weighted population estimates that allow for the complex survey design, this sample weighted to an estimated population of 5,180 (95% CI: 5,130–5,180). Of the sample youth 50.4% (95% CI 45.8, 55) were males, and 38.1% (95% CI 33.7, 42.9) were aged 12–13 years, 35.5% (95% CI 31.1, 39.9) 14–15 years, and 26.4% (95% CI 22.8, 30.4) 16–17 years. Around one-third reported smoking more than just a once or twice occurrence (34.7%, 95% CI 30.3, 39.5). These Aboriginal youth lived in more socioeconomically disadvantaged neighborhoods, with around 30% living in the bottom 10% of neighborhoods ranked by SES characteristics and less than 10% of their primary carers having 13 or more years of formal education. Further, one-fifth (20.7%) of their primary carers reported 7–14 family life stress events occurring in the 12 months prior to the survey.

## Measures

### Dependent variables

#### Lifetime health problems

This is a binary measure (1 = none, 2 = one or more lifetime health problems) derived from the sum of primary carer reports of youth ever having experienced one or more of 19 health problems e.g., epilepsy, kidney/renal disease, arthritis/rheumatism, developmental delay, muscular dystrophy, recurring chest, ear and/or skin infections, allergies. As this index counts data internal consistency is not reported.

#### Asthma symptoms

This binary variable measures asthma and associated respiratory symptoms using three survey questions developed for the International Study of Asthma and Allergies in Childhood (ISAAC)[53]. Youth self-reports were coded as having an asthma symptom if they responded ‘yes’ to one or more of the following questions: have you ever had asthma, in the past 12 months has your chest ever sounded wheezy during or after exercise, and in the past 12 months have you ever taken any medication (medicines, pills, puffers) for wheezing or asthma.

### Independent Variables

#### Defining psychosocial resilient status

This primary variable of interest was derived by grouping youths according to their family level risk exposure (high/low) and psychosocial functioning (good/poor) [29] using a 2 x 2 full classification model [55], and is briefly described below.

*Family-level risk*. Five risk factors were previously identified as specific predictors of increased likelihood of poor psychosocial functioning amongst Aboriginal youth [56]. The five measures comprise (a) *low nurturing parenting;* (b) *harsh parenting*; (c) *exposure to family violence*; (d) *single parent household*; and (e) *unemployed primary carer*. High family risk was defined as exposure to 2 or more risks.

*Psychosocial functioning*. This was measured from responses to the Youth Self Report form of Goodman’s Strengths and Difficulties Questionnaire (SDQ, [57]). Extensive pilot-testing and modeling of the SDQ subscales was undertaken for the WAACHS to confirm its reliability and validity for use with a diverse population of Aboriginal families and the 20-item scale showed good reliability of 0.93 [58]. SDQ cut points were defined as: “good”, 0–15 (normal range), and “poor”, 16–40 (borderline/abnormal).

Four groups comprise psychosocial resilient status: Expected Good (low risk, good psychosocial functioning), Vulnerable (low risk, poor psychosocial functioning), Resilient (high risk, good psychosocial functioning), Less Resilient (high risk, poor psychosocial functioning).

#### Childhood factors

*Proportion of optimal birth weight* (POBW) is a ratio of actual birth weight and expected birth weight, taking into account a number of factors known to influence fetal growth rate e.g., gestational age, child sex, mother’s height. It utilizes youth birth record data drawn from the Western Australian Midwives Notification of Births database and is described fully in the WAACHS [53]. **Whether breastfed**: Primary carers were asked whether the young person was ever breastfed (“yes”/”no”).

#### Young person factors

Sex and age (“12–13 years”, “14–15 years”, and “16–17 years”) of youth are reported in categories. Youth smoked: Youth were asked whether they smoked cigarettes more than just once or twice (“yes”/”no”). Psychosocial resilient status is a derived 4-part categorical variable measuring psychosocial functioning in contexts of high and low family risk exposure empirically associated with poor psychosocial functioning [56]. The four groups of youth are those are those described in our previous research [29] and their profiles are briefly outlined in Table 1.

#### Family factors

Primary carer level of formal education was measured on a 5-part ordinal scale from primary carer response and recoded for this study to a 3-level variable (“less than 9 years”, “10–12 years”, and “13 or more years”). Index of poor housing quality is based on primary carer reports of whether their house meets eight indicators of healthy living practices, such as, washing children and clothes, waste removal, healthy food preparation, and separation of animals from people (i.e., screen doors). The scale ranges from “0” to “3 or more” with higher scores reflecting more indicators of poor housing and is discussed fully elsewhere [54]. A parent smokes is a binary variable (“no”/”yes”) of youth reporting whether they have a parent who smokes. Life Stress Events is a measure of the number of life stress events occurring in the family in the 12 months prior to the survey (carer report). Dichotomous (“no”/”yes”) responses to fourteen items (including death, sickness, overcrowding, unemployment, incarceration, and financial stress) were summed and a quartile split imposed (from “low” 0–2 to “high” 7+).

#### Neighborhood factors

Socio-Economic Index for Areas (SEIFA) is calculated from census data and indexes relative socio-economic disadvantage for each census district in Australia [51]. As the majority of Aboriginal children live in families in the bottom 50% of SEIFA neighborhoods were classified into a three-part variable of socioeconomic disadvantage from the lowest (10% most disadvantaged) to highest (top 50% or least disadvantaged) socioeconomically disadvantaged neighborhoods (“bottom 10%”, “10–50%” and “highest 50%”). Neighborhood problems is a 3-part variable based on carer endorsement of a checklist of 17 problems in their neighborhood/community, including vandalism, car stealing, family violence, drug and alcohol abuse, unemployment, racism, youth gangs. Responses were summed and a tertile split created where “low” = 0–2, “moderate” = 3–8, and “high” = 9–16. Youth self-reported safety uses six items from the WAACHS measuring feelings of safety at home, on public transport and in the community during the day or night. Responses were scored on a 5-point scale, where lower scores reflect lower feelings of safety, and summed across all items. Total scores ranging from 4 to 30 were then ranked into quartiles from “most unsafe” to “most safe”.

### Data analysis

The WAACHS sample was selected in three stages: census collection districts (CDs), families, and children. CDs were selected with the probability of inclusion proportional to the number of Aboriginal and Torres Strait Islander children living in the CD. As a result multi-level modelling was used to account for the hierarchical structure of the survey data. Hierarchical logistic regression modeling was used to compare the influence of multilevel predictors on the likelihood of (a) no lifetime health problems and (b) no symptoms of asthma.

Entering independent variables simultaneously in logistic regression models identifies the unique association of each single variable with a dependent variable when multiple and multilevel independent variables are thought to be interrelated. Thus logistic regression modelling determines the unique effect of a single variable independent of the effect of other variables in the model [54]. SAS version 9.2 was used for all analyses (SAS Institute Inc., Cary, NC, USA, 2000–2008). Reported associations between the outcome variables and the predictor variables are expressed as odds ratios. Odds ratios of less than 1.0 denote a reduced likelihood of the outcome relative to the reference category, and odds ratios of greater than 1.0 an increased likelihood of the outcome relative to the reference category [59, 60].

Because multicollinearity can result in non-convergence of logistic regression models [60] the selection of predictor variables in the models required careful consideration. As described above, psychosocial resilient status, an independent variable in this study, utilizes five family risk variables (harsh parenting, low nurturing parenting, exposure to family violence, sole parent status and unemployed primary carer). Remaining predictors were selected to pay respect to this composite variable. The logistic regression reference category for psychosocial resilient status was designated as Less Resilient as the primary comparison of interest was within high risk exposed youth to assess the extent to which processes underlying Resilient status protected against lifetime health problems and asthma symptoms.

## Results

The sample comprised four groups of psychosocial resilient status youth: Expected Good (41.3%, 95% CI 36.6, 46.2), Resilient (26.4%, 95% CI 22.6, 30.7), Less Resilient (21.4%, 95% CI 17.8, 25.5) and Vulnerable (10.9%, 95% CI 8.4, 13.8). Just over one-third (34.2%, 95% CI 29.8, 38.6%) of youth was reported by primary carers to have had no lifetime health problems. A close to significantly higher proportion of Resilient youth (39.0%, 95% CI 30.7, 47.5%) than Less Resilient youth (23.9%, 95% CI 17.0, 32.7%) had no lifetime health problems.

Nearly half the sample of youth or 49.5% (95% CI 44.9, 54.3%) self-reported no asthma symptoms. Bivariate analyses revealed no significant differences between youth with or without asthma symptoms by any of the predictor variables. Of particular interest was the non-significant association of youth smoking and asthma. Of youth who smoked, 56.9% (95% CI 48.6, 64.7) reported one or more asthma symptoms compared to 47% (95% CI 41.2, 52.7) of non-smoking youth. Significant differences are identified at the p = .05 level by 95% confidence intervals that do not overlap (see Table 2).

**Table 2:** Youth, family and neighborhood characteristics by lifetime health problems and asthma symptoms (n = 5180 95% CI 5130, 5180).

	LTHP—None34.2% (95% CI 29.8, 38.6)	Asthma symptoms—None49.5% (95% CI 44.9, 54.3)
**Childhood factors**		
POBW		
<85%	27.2 (17.4, 38.6)	48.2 (36.4, 60.8)
> = 85%	35.9 (30.6, 41.3)	50.6 (44.9, 56.3)
Breastfed?		
yes	32.7 (27.5, 38.4)	49.3 (43.6, 55.1)
no	32.5 (21.0, 46.3)	60.3 (46.1, 74.2)
not applicable^1^		43.8 (34.5, 54.3)
**Youth factors**		
Male	32.5 (26.7, 38.4)	48.5 (42.1, 54.8)
Female	35.9 (29.9, 42.7)	50.6 (43.7, 57.2)
Age(years)		
12–13	34.4 (27.0, 42.4)	52.4 (44.2, 60.0)
14–15	35.5 (28.6, 43.0)	48.2 (40.1, 56.2)
16–17	32.0 (25.1, 40.2)	47.2 (38.9, 55.3)
Youth:		
smoker	33.1 (28.0, 38.7)	53.0 (47.3, 58.8)
non-smoker	36.2 (29.0, 44.3)	43.1 (35.3, 51.4)
Psychosocial Resilient Status		
Expected Good	37.5 (30.2, 45.0)	56.5 (49.0, 63.8)
Resilient	39.0 (30.7, 47.5)	51.6 (42.7, 60.2)
Vulnerable	29.8 (19.6, 42.9)	43.1 (29.6, 55.9)
Less Resilient	23.9 (17.0, 32.7)	36.9 (27.3, 46.8)
Youth safety (quartiles)		
1 = unsafe	33.5 (26.2, 42.1)	49.2 (41.1, 57.6)
2	28.3 (20.8, 36.5)	52.6 (43.6, 62.2)
3	36.8 (28.0, 46.4)	47.0 (37.2, 57.2)
4 = safe	38.0 (28.5, 48.0)	49.6 (39.6, 59.5)
**Family factors**		
Primary carer education		
13+ years	28.3 (13.8, 50.2)	50.9 (32.4, 67.6)
10–12 years	33.0 (28.2, 38.4)	49.9 (44.3, 55.7)
9 years or less	39.9 (30.3, 49.9)	48.0 (38.0, 58.0)
Parent		
smoker	30.6 (22.6, 40.0)	49.6 (44.3, 55.2)
non-smoker	35.7 (30.8, 40.8)	49.4 (40.1, 58.3)
C1 7+ life stress events		
0–2	40.5 (31.7, 50.1)	49.0 (40.1, 58.3)
3–4	37.8 (28.8, 46.8)	46.6 (37.0, 55.6)
5–6	30.1 (22.1, 38.7)	53.7 (43.9, 63.0)
7–14	26.2 (16.6, 37.2)	48.8 (37.6, 59.2)
Indicators of poor housing quality		
0	33.7 (26.6, 42.0)	51.2 (43.5, 58.7)
1	36.0 (28.5, 43.6)	54.0 (45.3, 62.1)
2	30.4 (19.6, 42.9)	42.4 (29.1, 55.9)
3	36.1 (26.2, 48.0)	44.3 (33.6, 54.8)
**Neighborhood factors**		
SEIFA		
Bottom 10%	36.9 (28.9, 45.2)	51.1 (42.2, 60.1)
10–50%	32.0 (26.8, 37.6)	47.2 (41.5, 53.0)
Highest 50%	39.6 (21.5, 59.4)	59.2 (38.8, 77.6)
Community problems		
0–2	46.7 (37.4, 56.0)	46.7 (37.4, 56.0)
3–8	53.2 (45.0, 61.6)	53.2 (45.0, 61.6)
9–16	49.0 (41.9, 56.6)	49.0 (41.9, 56.6)

^1^ Not applicable = primary carer not birth mother; CI = confidence interval; YSR = Youth self-report; POBW = proportion of optimal birthweight; SEIFA = socioeconomic index for areas where bottom 10% is most disadvantaged.

### Predicting the Likelihood of Lifetime Physical Health Problems

The first logistic regression model used simultaneous entry of 13 independent variables on the likelihood of youth having no lifetime health problems (see Table 3).

**Table 3:** Modeling^1^ the relative effect of Psychosocial Resilient Status on Physical Health Status of 12–17 year old Aboriginal Youth (N = 5180, 95% CI 5130, 5180)

	No Lifetime Health ProblemsModel 1	No Asthma SymptomsModel 2
	OR (p-value)	OR (p-value)
Psychosocial Resilient Status		
Expected Good	**2.82** (0.001)	**2.47** (<.001)
Resilient	**3.48** (<.001)	**1.76** (0.040)
Vulnerable	2.33 (0.052)	1.34 (0.384)
Less Resilient	Ref.	Ref.
YSR safety (quartiles)		
1 most unsafe	Ref.	Ref.
2	**0.46** (0.015)	0.89 (0.669)
3	0.80 (0.446)	0.77 (0.318)
4 most safe	0.97 (0.931)	0.87 (0.608)
Life stress events		
0–2	**2.22** (0.047)	1.01 (0.963)
3–4	1.39 (0.380)	0.87 (0.619)
5–6	1.39 (0.402)	1.23 (0.445)
7–14	Ref.	Ref.

^1^Table does not show tested explanatory variables with p-values > 0.10

This yielded three independent and significant relationships. In line with our hypothesis, the derived psychosocial resilient status variable was significantly and independently related to lifetime health problems. Specifically, Resilient (OR = 3.48; *p*<.001) and Expected Good (OR = 2.82; *p* = .001) youth were more likely than Less Resilient youth (ref) to have no primary carer reported lifetime health problems. Carers of youth exposed to low (0–2) life stress events were more than twice as likely to report no lifetime health problems (OR 2.22, p<.05) than those youth exposed to high (7+) life stress events. Youth self-reported feelings of being unsafe were associated with a lower likelihood of having no lifetime health problems, although this relationship was neither consistent nor strong.

### Predicting the Likelihood of Self-Reported Asthma Symptoms

The only statistically significant predictor in this model was psychosocial resilient status. Expected Good and Resilient youth were significantly more likely to report no asthma symptoms than Less Resilient youth (OR 2.47, p<.001, and OR 1.76, p<.04 respectively, see Table 3).

## Discussion

This study investigated links between psychosocial resilient status, multiple environmental risks, lifetime health problems and asthma symptoms in an urban sample of Aboriginal youth aged 12–17 years. The primary finding supported our principle hypothesis that psychosocial resilient status would be independently and significantly associated with both carer reported lifetime health problems and youth self-reports of asthma symptoms. Both Expected Good and Resilient youth were significantly less likely to have lifetime health problems and asthma symptoms than Less Resilient youth independent of a range of other plausible influences. This finding provides preliminary evidence that interventions and supports that foster the mental wellbeing among at-risk Aboriginal youth may also confer benefits to their physical health. The primary study finding is also consistent with biopsychosocial models of accumulating risks and resources collectively impacting on mental and physical health [23, 61]. It confirms that even within a large sample of Western Australian Aboriginal youth there are unique patterns of risks, resources and adaptation that can differentially impact on physical health.

### Psychosocial resilient status: social connections and physical health

Positive social connections underlying the psychosocial resilient status derived measure were significantly protective only for high family risk exposed youth influencing not only their psychosocial functioning but physical health. In high family risk contexts, Resilient youth were nearly 3.5 times as likely as Less Resilient youth to have no lifetime health problems (primary carer reported) and nearly twice as likely to report no asthma symptoms (self-reported). The importance of supportive social connections on positive developmental outcomes of children living with adversity is one of the most consistent findings in the resilience literature [62].

The limited resilience research with Indigenous youth and their communities similarly highlights the singular importance of social support [63]. In Australia, it is increasingly recognized that engagement of Aboriginal children in community-based natural resource management programs also benefits their identity development, cultural and social connectedness. These programs use culturally-relevant, land and sea management activities, and in the process expose young people to positive adult role models and provide opportunities for engagement with prosocial peers [64–66]. Participation in such natural resource management activities has been associated with multiple benefits including a reduction in perceived stress and indicators of diabetes and cardiovascular disease [67, 68].

Our results suggest that, for urban high family risk exposed Resilient youth, the protective influence of positive social connections afforded by living in low SES neighborhoods (inhabited by the majority of the Aboriginal population) may facilitate ready access to extended family support, in contrast to those youth living in higher SES neighborhoods. Aboriginal youth in upward socially mobile families may not only be exposed to actual discrimination but feel a very identifiable minority with attendant increase in stress [56, 69]. Therefore, government policies aimed at facilitating upward socioeconomic mobility may unintentionally impose sufficient burden to lower individual adaptive functioning for Aboriginal youth.

Despite the absence of biomarkers of allostatic load it is plausible that the presence of social connections ameliorates the impact of high risk family environments by dampening precisely those physiological response mechanisms involved in conferring risk [40, 70]. Further research utilizing a resilience framework and data-linkage processes could examine the nexus between psychosocial and physical health among Aboriginal youth across time.

### Psychosocial resilient status: perceived racism and physical health

Perceived racism is widely recognized as a significant stressor and has been linked to psychosocial functioning and physical health among Indigenous peoples and African American populations [25, 71]. Perceived racism was a significant predictor of psychosocial function only for Aboriginal youth in low family risk contexts (Expected Good and Vulnerable groups of youth). Within the low risk exposed Aboriginal youth, Vulnerable youth were more likely than the Expected Good group to report exposure to racism [29]. According to some biopsychosocial models, cognitive appraisal and negative responses to racism may underlie the impact of self-reported racism on psychosocial function and physical health [72]. The anticipation of being treated differently because of racial group membership, and the consequences for coping strategies invoked, can depend on a number of factors including preparation for bias and relevance of an event to self-identity [73, 74].

Finally, although multilevel variables at the individual, family and community level of influence were modelled, it is noteworthy that few variables other than psychosocial resilient status were independently associated with lifetime health problems or asthma symptoms. One explanation may lie in the comprehensive process undertaken to assess risks and resources from which the psychosocial resilient status variable was derived [29, 45]. For example, whereas other research has found factors related to cultural identity and continuity at the family and individual level to be related to psychosocial function and youth suicide e.g., [75, 76] our previous research found an inverse relationship [56]. This research with a sample of high family risk exposed Aboriginal youth from urban and remote regions of Western Australia, utilised proxy measures of cultural continuity, such as knowledge of Indigenous culture and language, or being in families impacted by policies of forced removals of children from families and homelands. Even including youth residing in remote locations where cultural measures such as speaking an Aboriginal language are stronger did not yield significant relationships in the expected direction and as such they were not included in these current analyses [56]. Despite the lack of statistical significance, it is important to acknowledge the past colonial policies of welfare and assimilation which separated Aboriginal children from families and Aboriginal families from their traditional lands, and the complexities involved in empirically measuring the consequences for current generations living with the feelings of loss, trauma and anger [2, 77, 78].

The current study contributes a rare empirical examination of associations between the psychosocial resilience and physical health of urban Aboriginal youth. It begins to tease out the complex relationship between contextually specific risks and resources, psychosocial wellbeing and physical health within a widely recognized disadvantaged, low SES, sample of urban Aboriginal youth. The continuing mental and physical health disparities between Australian Aboriginal and non-Aboriginal populations requires concerted attention be paid to the social processes and structural conditions perpetuating these inequalities. The study addresses an identified gap in the extant literature on physical health of urban Aboriginal youth and has provided preliminary evidence of factors buffering and exacerbating the impact of adverse social contexts. Critically, this research emphasizes that the needs of high risk youth are not the same as low risk youth and interventions need to be targeted accordingly.

## Strengths and Limitations

This study addresses a number of gaps in the extant and limited literature pertaining to urban Aboriginal youth. It contributes new evidence of associations between unique patterns of risks, resources, and psychosocial resilience and is the first study, to our knowledge, that investigates these relationships *within* an urban sample of Aboriginal youth in relation to physical health. This serves to highlight the heterogeneity of contexts and diversity of responses to similar high family risk environments within this circumscribed subset of the Aboriginal population. As such, the results may not be generalizable to Western Australian Aboriginal youth in geographically rural or remote environments nor to Indigenous youth elsewhere.

The WAACHS was not specifically designed to support an empirical examination of psychosocial resilience and physical health among Aboriginal youth. Respondent fatigue, time and cost of administering face to face questionnaires across a geographically dispersed population was a factor determining the extent to which psychological processes and physical health assessments could be assessed. Therefore some of the measures utilized in this current study can only be considered blunt indicators of physical health and wellbeing. While the data source is now over ten years old, they still provide a reliable assessment of the social, economic and health circumstances of Aboriginal children and families as there have been few significant changes in these circumstances across Australia since the WAACHS data were collected [1].

Asthma symptoms are self-reported by young people and there is some concern about the extent to which negative emotionality could systematically influence the self-reporting of physical health status [28, 79]. To address this issue we also use primary carer reports of youth lifetime health problems. Finally, given the complex etiology of asthma it was not possible to control for all possible confounders. Nevertheless, given the paucity of research on physical health in urban Aboriginal youth [37, 80] this current research provides insights into the heterogeneity of environmental exposures and sources of adaptation that ultimately may contribute to improved targeting of health prevention and intervention strategies.

## Conclusion

This study provides a more nuanced understanding of the multilevel influences impacting lifetime health problems and asthma symptoms in an urban sample of Australian Aboriginal youth. The composite construct of psychosocial resilient status has demonstrated that processes differentiating psychosocial functioning in both low family risk and high family risk contexts are also associated with less lifetime health problems and asthma symptoms. Thus the beneficial effects of a warm and nurturing family environment on a range of child developmental outcomes are unchallenged, constituting a fundamental and expectable environment of care [81]. Ensuring families have the capabilities to provide these caring and nurturing environments for children is a necessary step to maximising capability expansion within the Aboriginal population [82].

There is a broadly acknowledged link between toxic levels of stress and child developmental outcomes [15]. However, it is also clear that there is great heterogeneity in responses to similar risk exposures [11, 14, 83]. A more comprehensive representation of the risks and resources impacting the lives of Aboriginal youth, and the specific processes aiding their successful navigation of these challenges, is a step towards better targeting of prevention and intervention strategies to meet the complex needs of high risk youth.
